# Rare but Not Infrequent: Infective Endocarditis Caused by *Abiotrophia defectiva*

**DOI:** 10.1155/2018/5186520

**Published:** 2018-03-26

**Authors:** Sonia Chowdhury, Matthew L. German

**Affiliations:** Department of Internal Medicine, St. Luke's Hospital, Chesterfield, MO 63017, USA

## Abstract

Endocarditis (IE) is defined by an infection of a native or prosthetic heart valve, the mural endocardium, or an indwelling cardiac device. Although viridan-group streptococci (VGS) and *Staphylococci* species have collectively been considered as the most common cause of endocarditis, uncommon pathogens may also lead to the disease with significant morbidity and mortality. *Abiotrophia defectiva*, a nutritionally variant streptococci (NVS), is a virulent bacterium that preferentially affects endovascular structure and is implicated in many culture-negative endocarditis with dreadful complications such as heart failure, septic embolization, and valve destruction. Here, we report a case of a 60-year-old male patient, with a past medical history significant for hypertrophic obstructive cardiomyopathy, who was incidentally found to have mitral valve vegetative mass with an uncommon agent, *A. defectiva*. The patient was successfully treated with antimicrobial therapy. The objective of this article is to describe the possibility of uncommon cause of common diseases and raises awareness of infective endocarditis caused by *A. defectiva* among clinicians and microbiologists. Early and proper identification of this pathogen is important to achieve a better outcome.

## 1. Introduction

*Abiotrophia defectiva*, a variant of streptococci, was first identified approximately five decades ago. *A. defectiva* is part of the normal flora of the oral cavity and urogenital and intestinal tracts. This organism has been involved in various disease processes such as cerebral abscess, pancreatic abscess, corneal ulcer, sinusitis, osteomyelitis, and scrotal abscess. However, it is rare, yet not uncommon cause of infective endocarditis, and is estimated to cause approximately 4–6% of all streptococcal endocarditis with grave complications if not promptly identified and aggressively treated. Our case highlights the affinity of this agent to cause endocarditis, the delays in identification, and the necessity of more advanced microbiological diagnostic methods.

## 2. Case Presentation

A 60-year-old male, with a past medical history significant for hypertrophic obstructive cardiomyopathy, hypertension, and obstructive sleep apnea, was admitted to the hospital for evaluation of low-grade fever and fatigue that had started about 4 weeks before the hospital presentation. A rapid strep test (RST) on his throat was done, which came back positive for group A *β*-hemolytic streptococcus. The patient was treated with cephalexin 500 mg twice daily for ten days without any significant change in his symptoms and continued to have fever and chills. Subsequently, he went to an urgent care where routine blood work and chest X-ray were performed. Laboratory studies showed normal white blood cell (WBC) count and serum creatinine of 1.7 mg/dL (normal: 0.6 to 1.3 mg/dL), and chest X-ray was unremarkable. The patient was given azithromycin 500 mg daily for three days, but his symptoms did not improve.

Three days later, on July 25, 2017, blood cultures were obtained and grew a nutritionally deficient streptococcus, *Abiotrophia defectiva*. The patient did not have recent surgery or dental procedure, and a specific port of entry was not identified. Also, the patient did not have any signs of endocarditis. The patient was started on oral levofloxacin 500 mg daily, as an outpatient with improvement of symptoms but relapsed after completion of ten-day antibiotic therapy.

**Figure 1:**
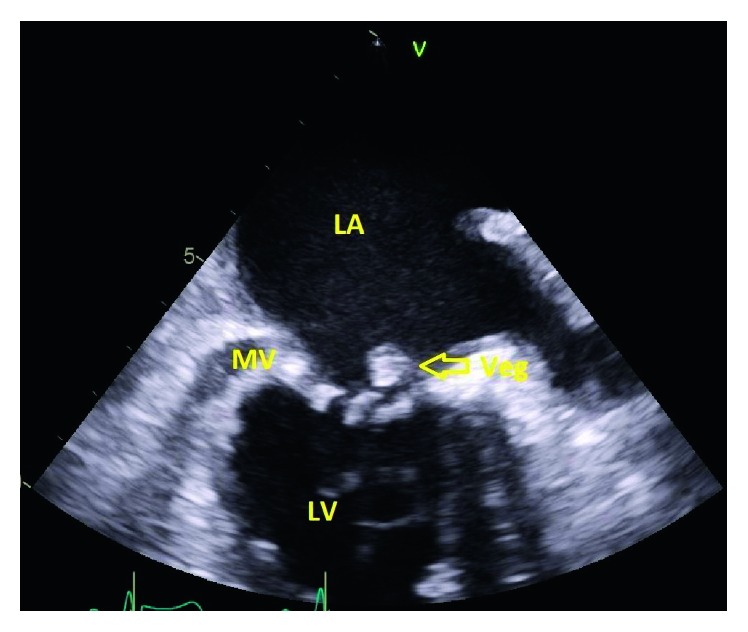
Transesophageal echocardiogram (TEE) showing a 0.63 cm × 0.54 cm vegetation on the posterior aspect of the mitral leaflet (indicated by arrow). LA: left atrium; MV: mitral valve; LV: left ventricle.

Finally, on August 11, 2017, the patient presented to the hospital for evaluation of his ongoing symptoms of low-grade fever, shortness of breath, fatigue, and approximately 5 pounds weight loss over one month. Physical examination revealed blood pressure of 125/79 mmHg and temperature of 37.0°C. Cardiovascular examination revealed a soft systolic murmur at the right upper sternal border and at the apex. The patient was alert and oriented without any focal neurologic deficit. Other physical examination was unremarkable. Laboratory studies on admission revealed blood urea nitrogen 21 mg/dL (normal: 7 to 20 mg/dL), chloride 108 mmol/L (normal: 96 to 106 mmol/L), hemoglobin 9.4 g/dL (normal: 13–18 g/dL), C-reactive protein 2.3 mg/dL, and estimated glomerular filtration rate (eGFR) 59 mL/min/1.73 m^2^. Three sets of blood cultures obtained on admission showed microscopic, nonhemolytic colonies on blood agar plates. Gram stain revealed pleomorphic, Gram-positive cocci in chain. The organisms grew well on chocolate agar but poorly on 5% blood agar media after 48-hour incubation. Subsequently, the organism identified as *Abiotrophia defectiva* when satellitism was found after a single cross streak of *Staphylococcus aureus* was placed on the blood agar plate. The isolate was subsequently found to be sensitive to penicillin, ampicillin, amoxicillin-clavulanic acid, ceftriaxone, gentamicin, erythromycin, and vancomycin. The antibiograms of all the other isolates were the same. A transesophageal echocardiogram (TEE) was done that revealed vegetation on the posterior aspect of the mitral leaflet, measuring 0.63 cm × 0.54 cm (Figure 1). The patient was initially treated with ceftriaxone 2 g intravenously (IV) once daily and gentamicin 5 mg/kg (320 mg) once daily. However, three days later, gentamicin was discontinued secondary to acute renal injury, and his baseline serum creatinine increased from 1.3 mg/dL to 3.1 mg/dL. Subsequent blood cultures, two sets from hospital days 1, 2, 3, and 5, remain sterile. The patient completed 6 weeks of IV ceftriaxone with complete recovery of symptoms and is currently being followed at the clinic.

## 3. Discussion

In 1961, Frenkel and Hirsch first isolated a new type of fastidious, thiol-requiring, vitamin B6-dependent, pyridoxal-dependent, Gram-positive, satelliting cocci [1–5]. In 1989, Bouvet and colleagues reclassified this organism into *Streptococcus defectivus* and *Streptococcus adjacens*, based on the DNA-DNA hybridization studies [3–5]. Later, in 1995, Kawamura and colleagues [5] created a new genus *Abiotrophia*, based on 16S rRNA gene sequence data and other phylogenetic analysis, and transferred these two species to the new genus as *Abiotrophia defectiva* and *Abiotrophia adiacens*. *A. defectiva* are classified as nonmotile, Gram-positive cocci in chains that are catalase negative and exhibit satellitism around colonies of other bacteria. *Abiotrophia* means “life nutrition deficiency” and refers to the species' requirements for supplemented media for growth. Due to the fastidious nutritional requirements, the organism grow either in the media enriched with pyridoxin and L-cystine, or in the absence of supplements, a streak of *Staphylococcus aureus* or *Staphylococcus epidermidis* provides suitable culture conditions, where these species grow as satellite colonies adjacent to the helper *Staphylococcus* species. *Abiotrophia* species are part of the normal microbiota of the oral cavity, but can also be found in the urogenital and gastrointestinal tracts, and can cause severe infections such as bacteremia, brain abscess, pancreatic abscess, meningitis, osteomyelitis, and in the rare occasion, endocarditis [1, 4, 6, 7]. Approximately, 5 to 6% of streptococcal endocarditis is caused by NVS [1, 4, 7]. Several studies have suggested the organism's higher affinity for endocardium due to the production of a significant amount of exopolysaccharide and propensity to bind with fibronectin in the extracellular matrix, which further expands their virulence [4, 6]. Preexisting valvular heart disease or other cardiac conditions are frequently associated with NVS-induced endocarditis [6]. Strict nutritional demand, fastidious nature, and delayed initiation of the appropriate antibiotic regimen have made this microbe a challenge to identify and treat.

*Abiotrophia defectiva* has displayed a notable tolerance to penicillin, and its minimum bactericidal concentration (MBC) of penicillin markedly exceeds the minimum inhibitory concentration (MBC/MIC), typically by 32-fold [8]. Antimicrobial susceptibility tests (ASTs) were performed on 132 clinical NVS, collected from 2008 to 2014, which revealed that 90% of the isolates have the higher level of penicillin resistance. However, *A. defectiva* is sensitive to vancomycin, ceftriaxone, and aminoglycoside [7]. A possible explanation of penicillin resistance is, under suboptimal nutritional conditional or if exposed to either penicillin or muralytic enzymes in a hypertonic medium, the organism produces L-forms that partially or entirely lack a cell wall, thus rendering antibiotics that target cell wall synthesis ineffective [1, 4, 9]. The American Heart Association (AHA) guidelines recommend that the treatment regimen used is the same as that used for *Enterococcus endocarditis* [10], that is, a combination of ampicillin or benzyl penicillin plus gentamicin for a period of 4–6 weeks. Alternatively, vancomycin can be used alone (without gentamicin) for six weeks for patients with penicillin allergy. Ceftriaxone combined with gentamicin may be an alternative treatment option for isolates with a penicillin MIC ≥0.5 *µ*g/mL that are susceptible to ceftriaxone [10].

## 4. Conclusion

In summary, this case illustrates an example of endocarditis by the rare but not uncommon pathogen, *Abiotrophia defectiva*. Clinicians should be aware of this condition which can easily be underestimated due to the fastidious nature and the challenge of recovering isolates from clinical specimens. Proper identification of this pathogen is important to prevent deadly cardiac complexity and hemodynamic compromise and for identification of other morbid signs, clinical course, and treatment. In conclusion, prompt diagnosis is essential to initiate the appropriate treatment as well as to prevent potentially fatal complications.
